# PMT6 Is Required for SWC4 in Positively Modulating Pepper Thermotolerance

**DOI:** 10.3390/ijms24054849

**Published:** 2023-03-02

**Authors:** Yu Huang, Weiwei Cai, Qiaoling Lu, Jingang Lv, Meiyun Wan, Deyi Guan, Sheng Yang, Shuilin He

**Affiliations:** 1 Key Laboratory of Applied Genetics of Universities in Fujian Province, Fujian Agriculture and Forestry University, Fuzhou 350002, China; 2 Agricultural College, Fujian Agriculture and Forestry University, Fuzhou 350002, China

**Keywords:** *Capsicum annuum*, PMT6, SWC4, heat stress, thermotolerance

## Abstract

High temperature stress (HTS), with growth and development impairment, is one of the most important abiotic stresses frequently encountered by plants, in particular solanacaes such as pepper, that mainly distribute in tropical and subtropical regions. Plants activate thermotolerance to cope with this stress; however, the underlying mechanism is currently not fully understood. SWC4, a shared component of SWR1- and NuA4 complexes implicated in chromatin remodeling, was previously found to be involved in the regulation of pepper thermotolerance, but the underlying mechanism remains poorly understood. Herein, PMT6, a putative methyltranferase was originally found to interact with SWC4 by co-immunoprecipitation (Co-IP)-combined LC/MS assay. This interaction was further confirmed by bimolecular fluorescent complimentary (BiFC) and Co-IP assay, and PMT6 was further found to confer SWC4 methylation. By virus-induced gene silencing, it was found that PMT6 silencing significantly reduced pepper basal thermotolerance and transcription of *CaHSP24* and significantly reduced the enrichment of chromatin-activation-related H3K9ac, H4K5ac, and H3K4me3 in TSS of *CaHSP24*, which was previously found to be positively regulated by CaSWC4. By contrast, the overexpression of PMT6 significantly enhanced basal thermotolerance of pepper plants. All these data indicate that PMT6 acts as a positive regulator in pepper thermotolerance, likely by methylating SWC4.

## 1. Introduction

Pepper (*Capsicum annuum*) is a solanacae with great agricultural importance. It is cultivated mainly in warm seasons in the tropical and subtropical regions and is frequently exposed to high temperature stress (HTS), with significant growth and development impairment [1,2]. The problem caused by HTS might become more serious due to the current global warming, and the most efficient countermeasure for this problem is to develop and utilize cultivars with high levels of thermotolerance, which might be improved by a better understanding of the mechanisms underlying thermotolerance [3,4]. According to previous studies, substantial proteins, such as BAX inhibitor-1 [5], HSPs [6,7], HSL [8], Kinases [9,10], glycerol-3-phosphate acyltransferase [11], MLO [12], and transcription factors, such as WRKYs [1,13,14], bZIPs [2,15], NACs [16], and HSFs [17], upregulate and act positively in pepper defense to HTS, with the thermotolerance-related genes being transcriptionally regulated by some crucial transcription factors, indicating the crucial role of transcriptional regulation in thermotolerance; however, the underlying mechanisms remain to be elucidated.

The prerequisite for transcriptional activation of a given target gene that is generally packed into chromatin and extremely compacted in the nuclei is their accessibility by the transcriptional machineries. To achieve this accessibility, the compacted chromatins should be unwrapped via chromatin remodeling [18]. One of the most actively studied mechanism for chromatin remodeling is incorporation of the H2A variant, H2A.Z [19,20,21], that is regulated by the SWR1 complex [22,23,24,25]. The occupation of H2A.Z is related to both active and inactive transcription in a position-dependent manner [26]. Another important epigenetic mechanism related to gene transcription is histone covalent modification; the methylation on different lysine residues of histone tails H3 and the extent (mono, di-, and tri-) of methylation or acetylation of lysine residues of histone H3 or H4 are closely related to the chromatin conditions. For example, H3K4me2, H3K9me2, and H3K27me2 are associated with heterochromatic silencing and transcription suppression [27,28,29,30], while H3K4me3, H3K9me3, and H3K36me3 are associated with activation of gene transcription [30,31]. The histone lysine methylations have been found to be catalyzed by various histone methyltransferases [32], and their acetylations are generally mediated by acetyltransferase complexes, such as Nucleosome Acetyltransferase of H4 (NuA4) complex or histone acetyltransferases (HATs) [33,34]. For expression of given genes, the incorporation of H2A.Z and histone covalent modification need to be coordinated, and this coordination might be achieved via the interaction among CRCs and other epigenetic machineries, such as histone methyltransferases and acetyltransferase complexes [35,36,37,38]. Components, for example, SWC4 shared by SWR1-C and NuA4-C [39,40], might be crucial for this coordination. The SWR-c and NuA4-c have been found to play roles in regulation of plant development [23,41,42,43], flowering time regulation [42,43,44], stress response, such as thermotolerance [43,45], DNA repair [43] using SWC4 for DNA binding or for SWR1-c and NuA4-c, as well as transcription factor recruiting [24,46]. For example, CaSWC4 acted positively in pepper response to high-temperature–high-humidity (HTHH) stress by recruiting transcription factors, such as CaWRKY40 and CabZIP63, and also by promoting deposition of chromatin activation related H2A.Z, H3K9ac, H4K5ac, and H3K4me3 to thermotolerance-related target genes. In addition, SWC4 might also act independently of its roles in NuA4 and SWR1 complexes in positively regulating telomere length [46,47]. As a crucial regulator involved in multiple biological processes, SWC4 should be tightly regulated; however, whether and how SWC4 is regulated by other regulatory proteins remain to be elucidated.

Methylation of DNA, histone, and crucial proteins mediated by various methyltransferases is crucial for gene transcription and for protein to function properly. Compared with the well-established histone methylation mediated by methyltransferases, such as SET-domain group (SDG) [48,49,50], and DNA methylation mediated by enzymes collected as DNA methyltransferases [51], the methylation of various proteins is one of most important post-translational modifications (PTM), but the majority of related methyltransferases remain to be functionally investigated. These methyltransferases identified by transcriptome and proteomics are sometimes collectively termed as putative methyltransferases (PMT) [52]. In the present study, a putative methytransferase (CaPMT6) was found in pepper to interact with CaSWC4, and it phenocopied with CaSWC4 in activating pepper thermotolerance in a mutually indispensable manner. It was also found that CaSWC4 was methylated by CaPMT6, and these results indicate that CaPMT6 fulfills its function by methylating CaSWC4 during pepper response to HTS.

## 2. Results

### 2.1. The Sequence of CaPMT6 and Its Comparsion with Its Orthologs in Other Plant Species

**Figure 1:**
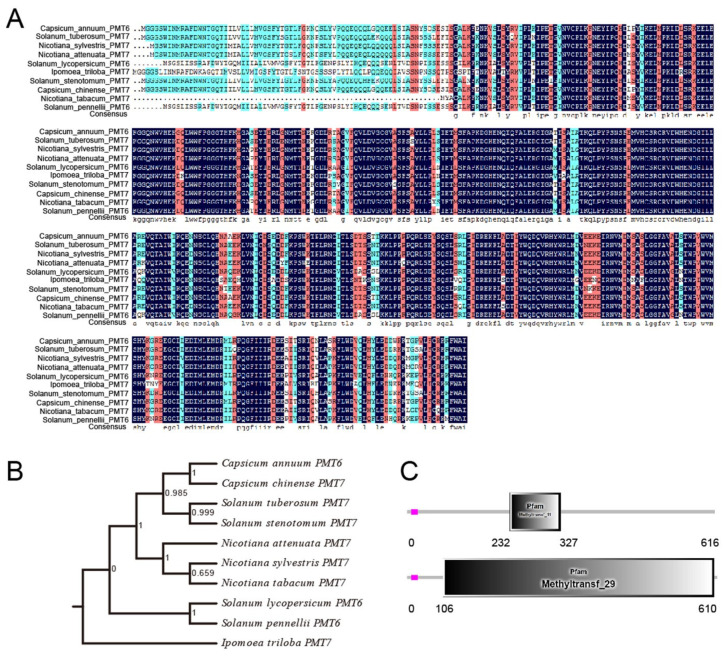
Analysis of amino acid sequence of pepper *CaPMT6* and the multiple alignment of CaPMT6 with its orthologues in other plant species. (**A**) Multiple alignment of amino acid sequences deduced from pepper *CaPMT6* with its orthologues from other plant species, including *Solanum tuberosum*, *Nicotiana sylvestris*, *Nicotiana attenuata*, *Solanum lycopersicum*, *Ipomoea triloba*, *Solanum stenotomum*, *Capsicum chinese*, *Nicotiana tabacum*, and *Solanum pennellii*. Blue shading, 50–75% identity; red shading, 75–100% identity; and black shading, 100% identity. (**B**) Phylogenetic analysis of *CaPMT6* with its orthologs in other plant species. (**C**) Two highly conserved methyltransferase domains in *CaPMT6* amino acid sequence.

In the potential interacting proteins of CaSWC4 identified by Co-IP combined with LC/MS, a putative methyltransferase aroused our interest: its deduced amino acid sequence exhibits a high degree of similarities to the putative PMT6 or PMT7 in other plant species, including *Solanum tuberosum*, *Nicotiana sylvestris*, *Nicotiana attenuate*, *Solanum lycopersicum*, *Ipomoea triloba*, *Sulanum stenotomum*, *Capsicum chinense*, *Nicotiana babacum*, and *Solanum pennellii* (Figure 1A,B), and a highly conserved methyltranferase domain was found in the deduced amino acid sequence of CaPMT6 (Figure 1C). Thus, we named it CaPMT6.

### 2.2. CaPMT6 Interacted with CaSWC4

**Figure 2:**
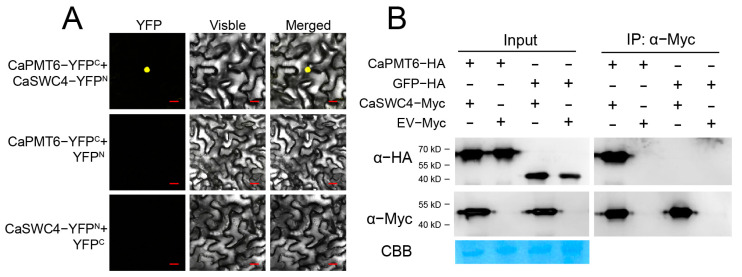
The interaction between CaPMT6 and CaSWC4. (**A**) CaPMT6 interacts with CaSWC4 by BiFC, CaPMT6 was fused to YFP^C^, and CaSWC4 to YFP^N^, which were co-transiently overexpressed in leaves of NB plants by agroinfiltration, and the YFP signal was detected under a laser scanning confocal microscopy at 48 hpi, Yellow fluorescence, visible light, and merged images were taken on a confocal microscope at 48 hpi. Bars = 25 μm. (**B**) Interaction between CaPMT6 and CaSWC4 in vivo, as determined by co-immunoprecipitation assay; proteins were isolated from pepper leaves transiently overexpressing CaPMT6-HA/CaSWC4-Myc, which were immunoprecipitated with anti-Myc antibody; and the presence of the tested interacting proteins was detected using antibody of HA by western blotting.

To confirm that CaPMT6 interacts with CaSWC4, we performed BiFC with the full-length open reading frame (ORF) of CaPMT6 fusing to YFP^C^ and ORF of CaSWC4 fusing to YFP^N^, and we found that CaPMT6 interacted with CaSWC4 in the nuclei by transient overexpression in the epidermal cells of *Nicotiana benthamiana* leaves (Figure 2A). This result was further confirmed by Co-IP assay using proteins isolated from CaSWC4-Myc and CaPMT6-HA transiently overexpressing pepper leaves; the CaSWC4-Myc was immunoprecipitated with antibody of Myc, the presence of CaPMT6 was detected by immune blotting using antibody of HA, and the result further showed that CaSWC4 interacted with CaPMT6 (Figure 2B).

### 2.3. CaPMT6 Was Upregulated by HTS

**Figure 3:**
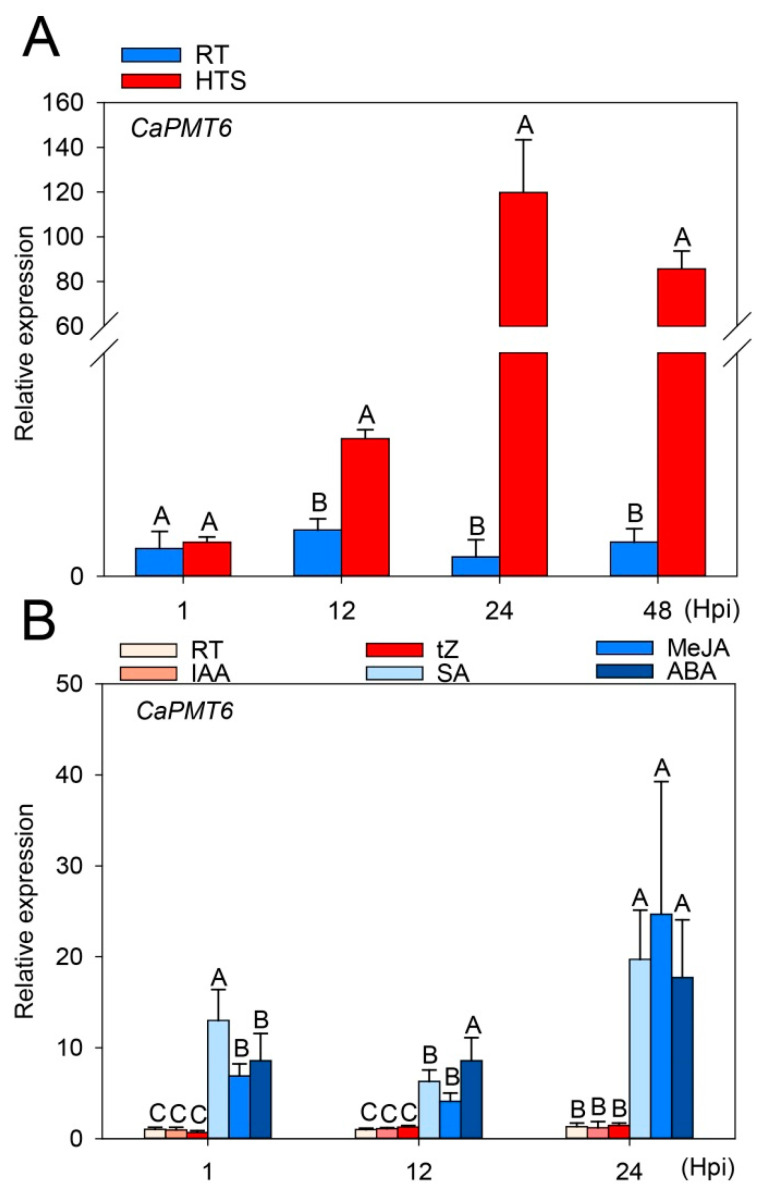
CaPMT6 was upregulated by HTS and by exogenous application of ABA. (**A**) The relative transcript level of CaPMT6 in pepper plants challenged by HTS. (**B**) The relative transcript level of CaPMT6 in pepper plants upon exogenously applied SA, MeJA, or ABA. In (**A**,**B**), *CaActin* was used as an internal control; data are shown as means ± standard error of six replicates; different capital letters above the bars indicated significant differences among means (*p* < 0.01).

By previous study, *CaSWC4* was upregulated by HTS and acts positively in pepper thermotolerance [46]. To assay whether *CaPMT6* is also involved in pepper response to HTS, the transcript level of *CaPMT6* was assayed in pepper plants challenged with HTS, and the result showed that *CaPMT6* was upregulated at transcriptional level from 12 to 48 hpt (hours post treatment). In parallel, the transcript level of *CaPMT6* in pepper plants challenged with exogenous application of SA, MeJA, or ABA were detected, and it was found that transcript level of *CaPMT6* increased upon exogenous application of SA, MeJA, or ABA (Figure 3). All these data indicate that *CaPMT6* might be involved in pepper defense response to HTS, likely in an ABA-signaling-dependent manner.

### 2.4. CaPMT6 Located in the Whole Cell, Including the Nuclei and Plasma Membrane

**Figure 4:**
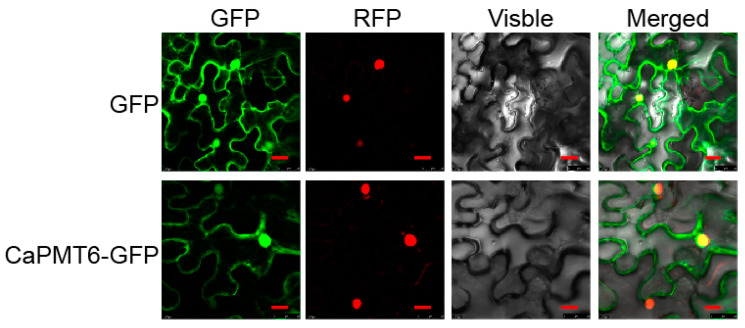
Subcellular location of CaPMT6 in epidermal cells of *N. benthamiana* leaves. *N. benthamiana* leaves were infiltrated with *Agrobacterium* GV3101 cells containing *35Spro:CaPMT6-GFP* (using *35Spro:GFP* as control); we used NbH2B (histone H2B)-RFP to indicate the nucleus. Subcellular localization of the CaPMT6-GFP fusion protein or control GFP was captured on a fluorescent confocal microscope at 48 hpi. Fluorescence images, bright-field images, and the corresponding overlay images of representative cells expressing GFP or CaPMT6-GFP fusion protein are shown; Bars = 50 µm.

As the function of a given protein is closely related to its subcellular location, we assayed the subcellular location of CaPMT6 by transient overexpression of CaPMT6-GFP in the epidermal cells of *N. benthamiana* leaves; the results showed that, similar to the control GFP, the GFP signal in CaPMT6-GFP transiently overexpressing leaves of *N. benthamiana* plants was found in the whole cell, including nucleus and plasma membrane (Figure 4), indicating that CaPMT6 might locate in the whole cell.

### 2.5. The Silencing of CaPMT6 Reduced Pepper Thermotolerance and Downregulated CaHSP24

**Figure 5:**
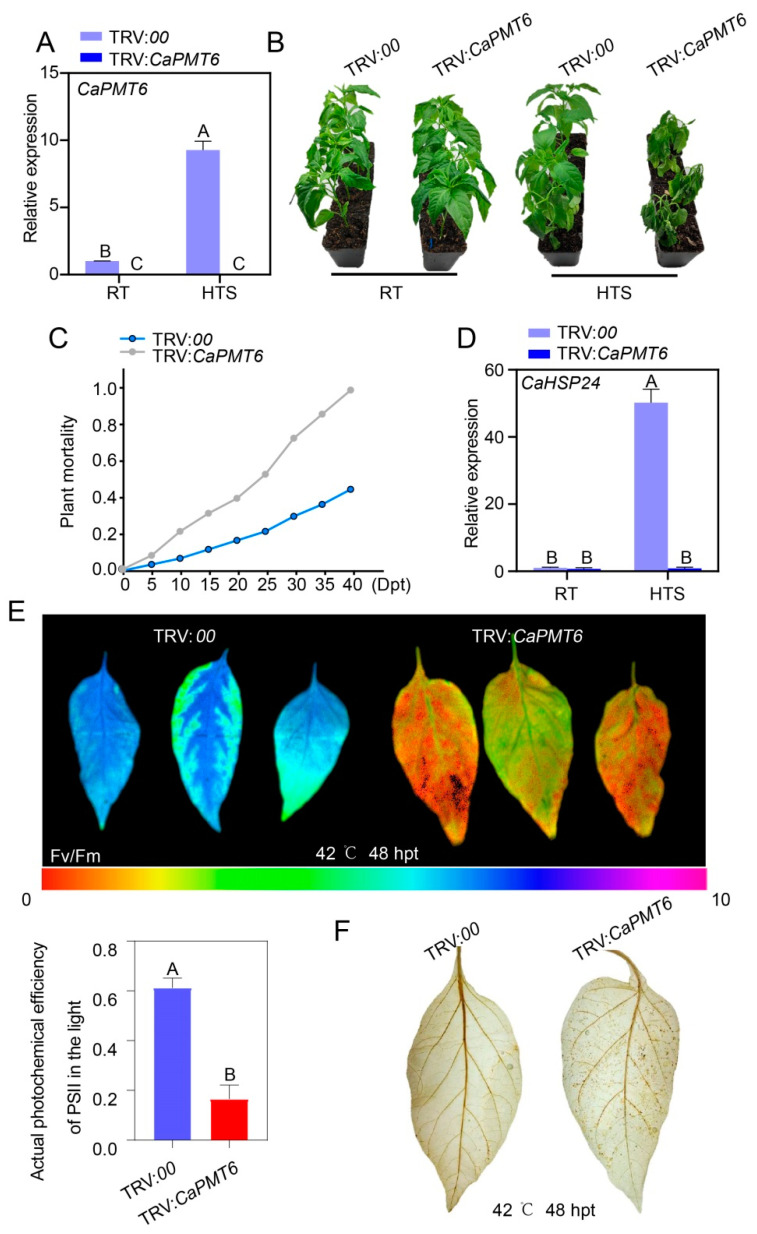
CaPMT6 silencing reduced pepper thermotolerance. (**A**) The success of *CaPMT6* silencing by virus-induced gene silencing (VIGS) by measuring the transcript level of *CaPMT6* in RT and HTS-challenged TRV:*CaPMT6* pepper plants at 24 h post treatment (hpt). The transcript levels of TRV:*00*/RT was set to 1. (**B**) TRV:*CaPMT6* pepper plants exhibited reduced thermotolerance compared with the wild type control plants. (**C**) TRV:*CaPMT6* pepper plants exhibited higher mortality upon HTS compared with the wild type plants; a total of 20 plants were calculated. (**D**) The relative transcript level of *CaHSP24* in TRV:*CaPMT6* pepper plants was significantly lower than that in TRV:*00* pepper plants; *CaActin* was used as an internal control; the transcript levels of TRV:*00*/RT was set to 1. (**E**) HTS-challenged TRV:*CaPMT6* pepper plants exhibited reduced optimal/maximal photochemical efficiency of PSII in the dark (Fv/Fm) and reduced actual photochemical efficiency of PSII in the light (Φ*PSII*) compared with TRV:*00* plants. (**F**) Higher level of H_2_O_2_ accumulation displayed by darker DAB staining was found in the TRV:*CaPMT6* pepper plants compared with TRV:*00* pepper plants. In (**A**,**D**,**E**), data are shown as means ± standard error of eight replicates; different capital lettersabove the bars indicated significant differences among means (*p* < 0.01), as calculated with *t*-test.

To functionally characterize *CaPMT6* in pepper plants, we generated *CaPMT6*-silencing pepper plants by virus-induced gene silencing (VIGS). To do this, a specific fragment in *CaPMT6* was employed for vector construction, and the fragment specificity was confirmed by sequence blast against the publicly available pepper genome sequence. No phenotypic difference was found between TRV:*CaPMT6* and TRV:*00* plants, but the transcript level of *CaPMT6* in HTS-challenged TRV:*CaPMT6* was less than 5% of that in the wild type control plants (TRV:*00* plants), indicating the success of *CaPMT6* silencing (Figure 5A,B). Upon HTS, a significant enhanced susceptibility to HTS was found in TRV:*CaPMT6* compared with TRV:*00* plants, and significant enhanced dynamic plant mortalities were found in TRV:*CaPMT6* plants (Figure 5C). In addition, reduced optimal/maximal photochemical efficiency of PSII in the dark (Fv/Fm) and actual photochemical efficiency of PSII in the light (Φ*PSII*), which are positively related to thermotolerance, were found in TRV:*CaPMT6* pepper plants, and a higher level of H_2_O_2_ accumulation displayed by DAB staining was found in TRV:*CaPMT6* pepper plants compared with the wild type plants (Figure 5E,F). Furthermore, the upregulation of *CaHSP24* by HTS in pepper plants was blocked by *CaPMT6* silencing (Figure 5D). All these data indicate that *CaPMT6* acts positively in pepper basal thermotolerance.

### 2.6. The Overexpression of CaPMT6-Potentiated Pepper Thermotolerance

**Figure 6:**
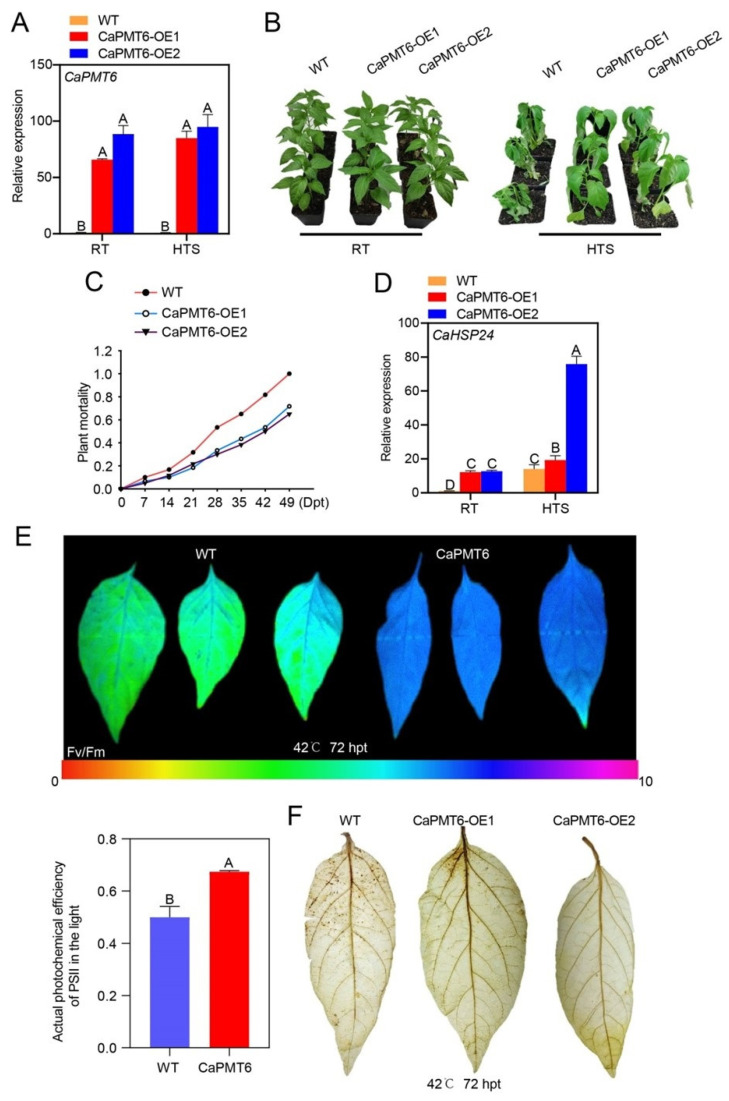
Overexpression of CaPMT6 significantly enhanced pepper thermotolerance. (**A**) The relative transcript level of *CaPMT6* in the two transgenic lines were significantly higher than that in the wild type pepper plants; *CaActin* was used as an internal control; the transcript levels of wild type/RT was set to 1. (**B**) The two CaPMT6 overexpressing pepper lines exhibited enhanced thermotolerance compared with the wild type plants. (**C**) *CaPMT6* overexpressing pepper plants exhibited higher mortality upon HTS compared with the wild type plants; a total of 20 plants were calculated. (**D**) The relative transcript level of *CaHSP24* in *CaPMT6*-overexpressing pepper plants was significantly higher than that in wild type pepper plants; *CaActin* was used as an internal control; the transcript levels of wild type/RT was set to 1. (**E**) HTS-challenged *CaPMT6*-overexpressing pepper plants exhibited increased optimal/maximal photochemical efficiency of PSII in the dark (Fv/Fm) and increased actual photochemical efficiency of PSII in the light (Φ*PSII*) compared with wild type pepper plants. (**F**) Lower level of H_2_O_2_ accumulation displayed by darker DAB staining was found in the *CaPMT6*-overexpressing pepper plants compared with the wild type pepper plants. In (**A**,**D**,**E**), data are shown as means ± standard error of eight replicates; different capital letters above the bars indicated significant differences among means (*p* < 0.01), as calculated with *t*-test.

To further confirm the role of *CaPMT6* as a positive regulator in pepper thermotolerance by VIGS, we generated *CaPMT6-GFP* overexpressing pepper plant lines; in total, more than 10 T_3_ lines were acquired, and two lines (CaPMT6-OE1 and -OE2) were randomly selected for further use (Figure 6A). A reduced susceptibility to HTS was found in both CaPMT6-OE1 and -OE2 compared with the wild type plants (Figure 6B). Consistently, reduced mortalities (Figure 6C) but enhanced Fv/Fm and Φ*PSII* values, as well as lower level of H_2_O_2_ accumulation, were detected in CaPMT6-OE1 and -OE2 compared with the wild type plants (Figure 6E,F). In addition, enhanced transcript level of *CaHSP24* was found in the transgenic plants compared with that in the wild type plants (Figure 6D). These data support the result from VIGS experiment that *CaPMT6* acts as positive regulator in pepper thermotolerance.

### 2.7. The Transient Overexpression of CaPMT6 Upregulated CaHSP24

In parallel, we performed agroinfiltration-based transient overexpression to study the possible role of CaPMT6 in pepper thermotolerance by detecting the effect of CaPMT6 transient overexpression on expression of thermotolerance-related *CaHSP24* and found that CaPMT6 was successfully expressed at mRNA and protein level (Figure S1A,B). We also found that the transient overexpression of CaPMT6 significantly upregulated *CaHSP24* (Figure S1C), supporting the notion that CaPMT6 acts positively in pepper thermotolerance.

### 2.8. CaSWC4 Is Required in Thermotolerance Activation by CaPMT6

**Figure 7:**
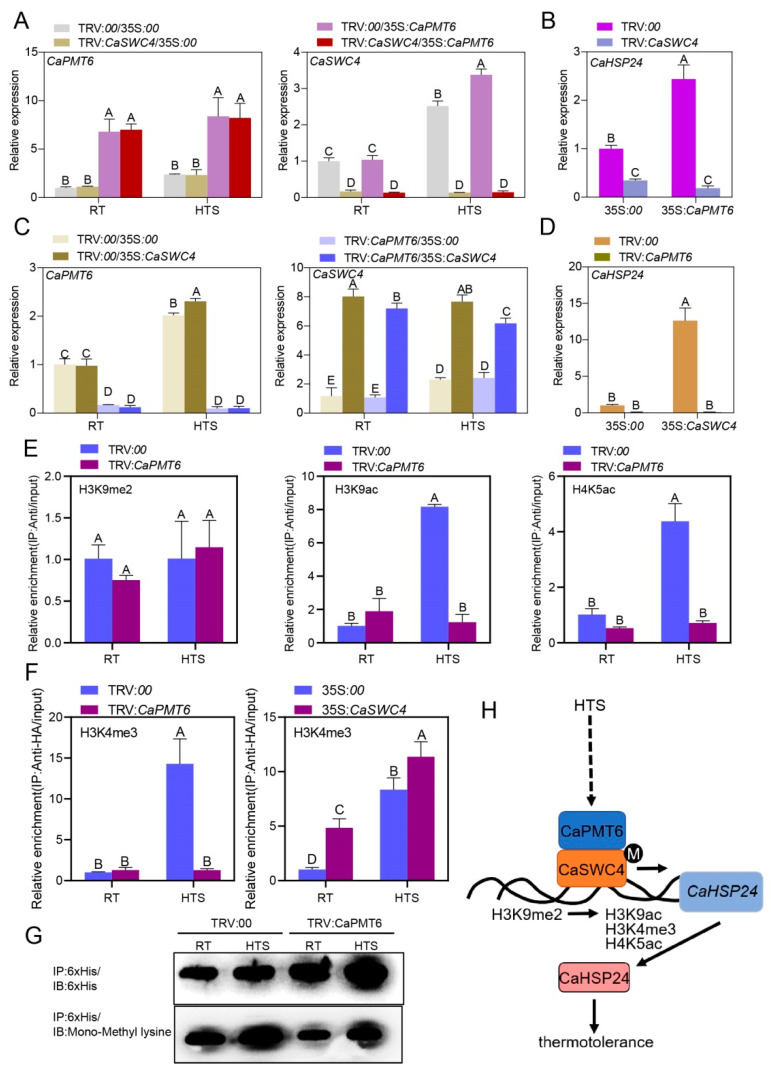
The functional relationship between CaPMT6 and CaSWC4 in modulating CaHSP24 expression. (**A**) The success of CaSWC4 silencing and transient overexpression of CaPMT6 by RT-qPCR in pepper plants. (**B**) The upregulation of CaHSP24 by transient overexpression of CaPMT6 was blocked by CaSWC4 silencing. (**C**) The success of CaPMT6 silencing and transient overexpression of CaSWC4 by RT-qPCR in pepper plants. (**D**) The upregulation of CaHSP24 by transient overexpression of CaSWC4 was blocked by CaPMT6 silencing. (**E**) The enrichment of H3K9ac and H4K5ac in the TSS of CaHSP24 was blocked by *CaPMT6* silencing upon HTS by ChIP-qPCR. (**F**) The enrichment of H3K4me3 in the TSS of *CaHSP24* by CaSWC4 transient overexpression upon HTS was blocked by *CaPMT6* silencing by ChIP-qPCR. (**G**) The effect of CaPMT6 silencing on methylation of CaSWC4 under different environmental conditions: CaSWC4-6 × His was expressed in *E.coli* and mixed with proteins isolated from TRV2:*CaPMT6* or wild type pepper plants under the conditions of RT (28 °C, 80% humidity) or HTS (42 °C, 80% humidity), and then CaSWC4-6 × His was immunoprecipitated with antibody of His, and the presence of CaSWC4-6 × His in the acquired protein was detected by immunoblotting with antibody of His, and the methylation of CaSWC4-6 × His was detected by immunoblotting using antibody of mono-methyl lysine. (**H**) Mechanism underlying CaHSP24 expression and, thus, tolerance to HTS mediated by CaSWC4-CaPMT6 module, CaPMT6 interacts with and methylates CaSWC4, thus turns on chromatin activation mediated by CaSWC4, and thus activates expression of *CaHSP24* and defense response to HTS. In (**A**,**D)**, *CaActin* was used as an internal control, the transcript levels of TRV:*00*/RT was set to 1; data are shown as means ± standard error of eight replicates; asterisks above the bars indicate significant differences among means (*p* < 0.01), as calculated with *t*-test. In (**E**,**F**), The enrichment levels of the tested genes were compared with those in the control, and the relative enrichment of IP using antibody/TRV:*00* was set to a value of 1 after normalization by input; data are shown as means ± standard error of three replicates. Different uppercase letters above the bars indicate significant differences between means (*p* < 0.01) by Fisher’s protected LSD test.

The CaSWC4-CaPMT6 interaction implied the functional relationship between CaSWC4 and CaPMT6. To confirm this speculation, we assayed their functional correlation during pepper response to HTS by silencing one gene and transiently overexpressing the other. The success of gene silencing and gene transient overexpression were confirmed by RT-qPCR (Figure 7A,C), and the result showed that *CaHSP24* was upregulated by transient overexpression of both CaSWC4 and CaPMT6. However, when CaSWC4 was silenced, the upregulation of *CaHSP24* by transient overexpression of CaPMT6 was blocked, and vice versa (Figure 7B,D). These data indicate that CaSWC4 and CaPMT6 are required by each other for their roles in pepper thermotolerance.

By previous study, the enrichment of chromatin-activation-related H3K4me3, H3K9ac, and H4K5ac in TSS of *CaHSP24* was found to be positively regulated by *CaSWC4*. The data in our present study showed that the enrichment of H3K9ac and H4K5ac in the TSS of *CaHSP24* was significantly blocked by the silencing of *CaPMT6* under the conditions of HTS; by contrast, the enrichment of chromatin-inactivation-related H3K9me2, which was reduced by transient overexpression of CaSWC4, and the enrichment of H3K9me2 in TSS of *CaHSP24* was not significantly affected by *CaPMT6* silencing (Figure 7E). In addition, the enrichment of chromatin-activation-related H3K9me3 in TSS of *CaHSP24*, which was increased by transient overexpression of CaSWC4, was significantly reduced by *CaPMT6* silencing upon HTS (Figure 7F). Importantly, the methylation level of CaSWC4 detected by immunoblotting with antibody of mono-methyl lysine was significantly reduced by *CaPMT6* silencing in RT- or HTS-treated pepper plants (Figure 7G). All these results indicate that upon HTS, CaSWC4 is methylated by methyltransferases, including CaPMT6. In this way, the chromatin activation of CaHSP24 and thermotolerance mediated by CaSWC4 is positively regulated by CaPMT6. (Figure 7H).

## 3. Discussion

The results of our previous study indicate that CaSWC4 acts positively in pepper thermotolerance, likely by coordinating SWR1- and NuA4-c in chromatin remodeling during activation of thermotolerance [46], but whether and how it is regulated by other regulatory proteins to fulfill its function has not been fully understood. The data in the present study indicate that CaPMT6 phenocopied with CaSWC4 in pepper response to HTS by physically interacting with and methylating CaSWC4.

### 3.1. CaPMT6 Is Regulated by HTS and Acts Positively in Pepper Thermotolerance

Our data showed that CaPMT6 was upregulated by HTS (Figure 3), implying that it might play a role in pepper thermotolerance since by previous studies, genes that upregulate in plants against a given stress might be involved in plant defense response to this stress [1,8,15]. This speculation was further confirmed by loss- and gain-of-function assay, in which *CaPMT6* silencing increased, while its overexpression reduced chemosensitivity, which displayed (by enhanced plant mortality) lower Fv/Fm and ΦPSII, which are positively related to thermotolerance [46]. The role of CaPMT6 as a positive regulator in pepper thermotolerance was closely related to lower level of H_2_O_2_ accumulation manifested by DAB staining and is consistent with the result of a previous study that accumulation of ROS, including H_2_O_2_, are negatively related to thermotolerance [53]. In addition, expression of *CaHSP24*, which contributes positively to thermotolerance [2,8,10,54], was found to be positively regulated by CaPMT6 (Figure 5D, Figure 6D, and Figure S1) and exogenous application of ABA, which has been found to be generally involved in regulation of plant thermotolerance [55,56], was found to upregulate *CaPMT6*. All these data support a role of CaPMT6 as a positive regulator in pepper thermotolerance in an ABA-signaling-dependent manner.

### 3.2. CaPMT6 Mediates Pepper Thermotolerance Probably by Methylating CaSWC4

SWC4 is a component shared by SWR1-c and NuA4-c [39,40], which orchestrates chromatin remodeling partially by using some of the shared components [23,44,46,57,58]. Our data also showed that *CaSWC4* silencing significantly reduced disposition of H2A.z, H3K9ac, H4K5ac, and H3K4me3 in the TSS of *CaHSP24*, supporting the result of our previous study that CaSWC4 [46] acts positively in pepper thermotolerance by chromatin activation through recruiting SWR1-, NuA4, and likely other histone methyltransferases, such as SET-domain group (SDG) family proteins [48,49,50,59]. This function of CaSWC4 appeared to be CaPMT6-dependent since the upregulation of *CaHSP24* as well as enrichment of H3K9ac, H4K5ac, and H3K4me3 in TSS of *CaHSP24* mediated by CaSWC4 transient overexpression [46] was blocked by *CaPMT6* silencing (Figure 7). Importantly, our data further indicate that CaPMT6 interacted with CaSWC4 (Figure 2), and this interaction might lead to enhanced methylation level of CaSWC4 (Figure 7G). We speculate that, unlike SDG proteins that methylate histone for chromatin remodeling, CaPMT6 might act positively in chromatin remodeling during pepper response to HTS by directly regulating CaSWC4 via methylation; however, to elucidate the molecular details underlying CaSWC4 methylation by CaPMT6, further study is required.

### 3.3. CaPMT6-CaSWC4 Module Might Also Be Involved in Pepper Immunity

Plant immunity and thermotolerance appear to be closely related; for example, WRKY25, -26 and -39 have been found to be employed by Arabidopsis not only in immunity but also in response to heat stress [60,61]. This phenomenon is more obvious in solanacaeous plants, including pepper, which has originated and evolved in subtropical and tropical regions that are frequently exposed to attacks from HTS and soil-borne pathogens, and a substantial number of genes, including CaCDPK15, CabZIP23, CabZIP63, CaWRKY6, CaWRKY40, and CaNAC2c [1,2,10,13,15,16,46,62,63], have been found to be employed in pepper to activate immunity and thermotolerance by interacting with other proteins post-translationally. This arrangement might benefit rapid transformation from defense response from one stress to another [15,46,64,65]. In addition to thermotolerance, CaSWC4 was previously found to act positively in pepper immunity against *Ralstonia solanacearum* infection [46]. The data in the present study indicate that CaPMT6 was not only upregulated by exogenous application of ABA but also upregulated by exogenous application of SA or MeJA (Figure 3), and signaling mediated by SA and JA has been generally found to be crucial for plant immunity, with SA signaling acting typically in plant immunity against biotrophic pathogens and JA signaling in resistance against necrotrophic pathogens [66,67,68]. Thus, it can be speculated that *CaPMT6* might also play a role in pepper immunity against infection of pathogens, such as *R. solanacearum* in a SA/JA-signaling-dependent manner. However, the role of CaPMT6 in pepper immunity remains to be confirmed, and how CaPMT6-CaSWC4 modules undergo post-translational modifications to context-specific activated thermotolerance and SA/JA-mediated immunity remain to be elucidated.

Collectively, the data in the present study indicate that CaPMT6 phenocopies and associates with CaSWC4 during pepper response to HTS, with CaSWC4 being methylated and, thus, its function in thermotolerance being regulated by CaPMT6.

## 4. Materials and Methods

### 4.1. Plant Materials and Growth Conditions

Seeds of pepper (*C. annuum*) inbred lines HN42 and *N. benthamiana* were sown in a soil mix (peat moss: perlite, 2:1 (*v*/*v*)) in plastic pots and placed in a growth room under at 25 °C, 60–70 µmol photons m^−2^ s^−1^, a relative humidity of 80%, and a 16 h light/8 h dark photoperiod.

### 4.2. Vector Construction

For vector construction, a Gateway cloning technique (Invitrogen, Carlsbad, CA, USA) and a series of Gateway-compatible destination vectors were employed. The full-length ORF of *CaPMT6* (*CaSWC4*) or its specific fragment of 300–500 bp in length within 3′UTR was cloned into pDONR207 by BP reaction; after confirmation by sequencing, the objective fragment was cloned into destination vectors, such as pEarleyGate103 (for overexpression) or TRV2 (for gene silencing by virus-induced gene silencing) by LR reaction, and resulting construct was further transformed into *Agrobacterium* GV3101 or *E. coli* DH5α for further use.

### 4.3. Agroinfiltration-Based Transient Overexpression in Leaves of Pepper and N. benthamiana Plant

GV3101 cells harboring *35S:CaPMT6-GFP* or *35S:GFP* vector were grown overnight in LB medium supplemented with appropriate antibiotics and then resuspended in induction medium (10 mM MES, 10 mM MgCl_2_, pH 5.4, and 150 µM acetosyringone) and adjusted to OD_600_ = 0.8. Then, 1mL of this bacterial suspension was injected into leaves of pepper plants at the eight-leaf stage or leaves of *N. benthaminana* using a syringe without a needle, and the injected leaves were harvested at 48 hpi (hours post injection) for further use. For subcellular localization assay, GFP signal was imaged at 48 hpi using a laser scanning confocal microscope (TCS SP8, Leica, Solms, Germany) with an excitation wavelength of 488 nm and a 505–530 nm bandpass emission filter.

### 4.4. Virus-Induced Gene Silencing (VIGS) Assay

GV3101 cells containing TRV2-*CaPMT6(CaSWC4)* and GV3101 cells containing TRV1 were resuspended in the induction medium at a 1:1 ratio; cells containing OD_600_ = 0.8, TRV2-*CaPMT6(CaSWC4),* and those containing TRV1 were mixed at 1:1 ratio, and approximately 300 μL of the mixture cells were infiltrated into cotyledons of 2-week-old pepper plants. The details of the process were as described in our previous studies [10].

### 4.5. The Application of HTS

The CaPMT6 or CaSWC4 silencing or overexpressing pepper seedlings of similar size and the wild type plants in pots were placed into an incubator under a condition of 42 °C, 60–70 µmol photons m^−2^ s^−1^, and a 16 h light/8 h dark photoperiod. To avoid any possible dehydration caused by the high temperature, sufficient water was applied to the plants with a more than 80% relative humidity.

### 4.6. Western Blot Assay

Western blotting was employed to detect the possible methylation and protein–protein interaction. To accomplish this, protein mixture dissolved in 5 × SDS loading buffer was denatured at 99 °C for 10 min and was then separated by SDS-PAGE gel. The gel was run at first under 80 V for 30 min and then 120 V for 40 min, and then the protein was transferred to PVDF membrane by semi-dry method under the condition of 200 mA/30 min. The membrane was then sealed with 5% skimmed milk powder TBST for 1h, and then the 1:5000 diluted appropriate first antibody was added and incubated at 4 °C overnight. TBST was used to clean the membrane three times at 25 °C, 80 rpm for 5 min. Then, 1:10,000 dissolved appropriate secondary antibody was added and incubated at 8 rpm, 25 °C for 1 h. Then, the membrane was cleaned with TBST three times at 80 rpm, 25 °C for 5 min. After cleaning, the membrane was fully applied with ECL chemiluminescence solution for 2 min, and then photographs were taken using Chemiluminescence imaging analyzer(LAS4000 imager, GE, Boston, MA, USA).

### 4.7. Chromatin Immunoprecipitation (ChIP)

ChIP assay was performed following the method of previous study [69]. Briefly, plants of *CaPMT6*-silencing pepper plants challenged with HTS were harvested at different time points, chromatins were isolated from leaves of these plants, which were crosslinked in a 1% formaldehyde solution, the chromatin was sheared into 300–500 bp fragments, and then DNA was immunoprecipitated with anti-H3k9ac, anti-H4K5ac, anti-H3k9me2, or anti-H3K9me3 (Abcam, Cambridge, UK). The DNAs acquired by immunoprecipitation with different antibodies were purified and used as templates for ChIP-qPCR using the specific primer pairs for ChIP-qPCR.

### 4.8. Bimolecular Fluorescent Complimentary (BiFC)

To confirm the interaction between CaPMT6 and CaSWC4 by BiFC, GV3101 cells containing construct *35S:CaPMT6-YFP^C^* and cells containing *35S:CaSWC4-YFP^N^* were mixed at 1:1 ratio, and the mixtures were infiltrated into leaves of *N. benthamiana* plants using *35S:CaPMT6-YFP^C^* + *35S:YFP^N^*; *35:YFP^C^ + 35S:CaSWC4-YFP^N^*, *35S:YFP^N^ + 35:YFP^C^* were used as negative controls. YFP fluorescence was imaged at 48 hpi using a laser scanning confocal microscope (TCS SP8, Leica, Solms, Germany) with an excitation wavelength of 488 nm and a 505–530 nm bandpass emission filter.

### 4.9. Co-Immunoprecipitation (Co-IP)

Leaves of *N. benthamiana* were co-infiltrated with GV3101 cells containing *35S:CaPMT6-YFP^C^-HA*, and cells *35S:CaSWC4-YFP^N^-Myc* were harvested at 48 hpi; total protein was extracted using protein extraction buffer (10% glycerol, 25 mM Tris-HCl, pH 7.5, 150 mM NaCl, 1 mM EDTA, 2% Triton X-100, 10 mM DTT, 1× complete protease inhibitor cocktail (Sigma-Aldrich, St. Louis, MO, USA), and 2% (*w*/*v*) PVPP). The acquired protein was incubated with monoclonal anti-HA magnetic beads (Sigma-Aldrich) at 4 °C overnight. Beads were then collected with a magnet and washed 3 times using protein extraction buffer. The eluted proteins were separated by SDS-PAGE electrophoresis and immunoblotted using anti-HA-peroxidase antibody or anti-Myc-peroxidase antibody (Sigma-Aldrich).

### 4.10. DAB Staining

The accumulation of H_2_O_2_ was assessed DAB by staining following the previously published method of Choi [1].

### 4.11. Chlorophyll Fluorescence Spectrophotometry

For thermotolerance assay, the *CaPMT6* silencing or transient overexpressing pepper or *N. benthamiana* plants were placed in an illumination incubator under the condition of 37 °C and 90% relative humidity. Fv/Fm and ΔF/Fm′ values of the plant leaves were measured using a MINI Imaging PAM instrument (Heinz Walz GmbH, Effeltrich, Germany). The plants were adapted to darkness for 15 min prior to chlorophyll fluorescence measurements being taken, following the method of Schreiber [70].

### 4.12. RT-qPCR

RT-qPCR was performed to detect the transcript levels of the selected genes as described previously [2]. A Bio-Rad real-time PCR system and SYBR PreMix Ex Taq II system were used. *CaActin* (GQ339766) was used as an internal reference gene, and the data were analyzed by the Livak method.

### 4.13. Development of Transgenic Pepper Plants

GV3101 cells containing *35S:CaPMT6-GFP* were suspended in suspension buffer (2 × MS, sucrose 30g /L, 1/5000 silwetL-77, 1/1000 AS, pH = 5.8) to OD_600_ = 0.4. To obtain transgenic pepper plants, flamingo bill method was used [71]. First, pepper seedlings were cultivated in a growth room until cotyledons fully expanded, then cotyledon was cut in half, and the damaged seedlings were then soaked in the suspension of GV3101 cells containing *35S*:*CaPMT6* for 13 min. The seedlings were transplanted back into the soil and the plants were placed in the growth room at 25 °C in the dark for 72 h. A 0.004% basta solution was applied to the damaged part of the seedlings at 3, 6, and 9 days post treatment (dpt) for screening. Thirty days later, new adventitious buds were observed, and these adventitious buds were cut when they grew to 2–3 cm and transplanted into soil supplemented with appropriate fertilizers under the conditions of 25 °C, 70% relative humidity, 16 h light/8 h darkness, and plants were developed from the adventitious buds within about one month. The transgenic plants were confirmed by PCR using specific primer pairs, and the transgenic plants were self-pollinated to obtain red fruits and the seeds of T_1_ lines. During their germination, the T1 seeds were further screened with 0.004% basta solution to obtain the T_1_ transgenic plants, which were also self-pollinated; the seeds of T_2_ or T_3_ lines were obtained a in similar way, and the plants of T_3_ lines were used for further assay.

### 4.14. Statistical Analyses

Statistical analyses were performed with the DPS software package. Data are shown as means ± SD obtained from three or four replicates; different capital letters indicate significant differences among means (*p* < 0.01), as calculated by Fisher’s protected least significant-difference (LSD) test.

## Data Availability

The data that supports the findings of this study are available in the supplementary material.

## Supplementary Materials

The following supporting information can be downloaded at: https://www.mdpi.com/article/10.3390/ijms24054849/s1.
