# Supplementary material for: PMT6 Is Required for SWC4 in Positively Modulating Pepper Thermotolerance
Source: Int J Mol Sci. 2023 Mar 2;24(5):4849. doi: 10.3390/ijms24054849 (PMC10003703; doi:10.3390/ijms24054849)
Supplement: Supplementary file 1 [file ijms-24-04849-s001.zip › ijms-2183217-supplementary.pdf]

Supplemental Table

Table S1. Primers used in this study.

	Gene	Forward primer	Reverse primer
**Primers used for *CaPMT6* study**	***CaPMT6***[1]	GGGGACAAGTTTGTACAAAAAAGCAGGCTTC<br>ATGGGTGGTGGATCTTGGAT	GGGGACCACTTTGTACAAGAAAGCTGGGTCAC<br>AAACATTTACTCCTGTTT
	***CaPMT6*-GFP**[2]	GGGGACAAGTTTGTACAAAAAAGCAGGCTTC<br>ATGGGTGGTGGATCTTGGAT	GGGGACCACTTTGTACAAGAAAGCTGGGTCTTA<br>AGCAATGGCCCAAAATC
	***CaPMT6*-VIGS**[3]	GGGGACAAGTTTGTACAAAAAAGCAGGCTTC<br>ATGGGTGGTGGATCTTGGAT	GGGGACCACTTTGTACAAGAAAGCTGGGTCAC<br>AAACATTTACTCCTGTTT
**Primers used for *CaSWC4* study**	***CaSWC4***	GGGGACAAGTTTGTACAAAAAAGCAGGCTTC<br>ATGGATGCGAAGGACATCTT	GGGGACCACTTTGTACAAGAAAGCTGGGTCTC<br>ATCCATCAGTCTTTAACT
	CaSWC4- GFP	GGGGACAAGTTTGTACAAAAAAGCAGGCTTC<br>ATGGATGCGAAGGACATCTT	GGGGACCACTTTGTACAAGAAAGCTGGGT<br>CTCCATCAGTCTTTAACTTGC
	CaSWC4-VIGS	GGGGACAAGTTTGTACAAAAAAGCAGGCTTC<br>ACTTCTTCTGCCATTGTGAT	GGGGACCACTTTGTACAAGAAAGCTGGGTCCC<br>ATCCTGGTAAGCAATCTAA
**Primers used for pepper q-PCR**	***CaPMT6*-qPCR**	GCGTCTCTGCTACTGCTGAA	AATCTTCTTGCCCTCCAGCC
	***CaSWC4*-qPCR**	TTGAGCGAAAACGTGCACTG	CAGGAGCAGCATCTGACACA
	***CaHSP24*-qPCR**	GTTCGTCTAGCAGTTTGGTTCGGTTG	GTAATTTAACTAAACAGACTCTTACAACC
	***CaACTIN*-qPCR**	AGGGATGGGTCAAAAGGATGC	GAGACAACACCGCCTGAATAGC
**Primer**	***PHSP24-Tss***	TGAGTTTCTCGAATCCTTTTTCCC	TTCGCCGACTTAGCTTCACG

[1]Primers used for ***CaPMT6*** full-length cloning

[2]Primers used for *35S:* ***CaPMT6***-*GFP* construct

[3]Primers used for TRV*:* ***CaPMT6*** construct

Supplemental figure

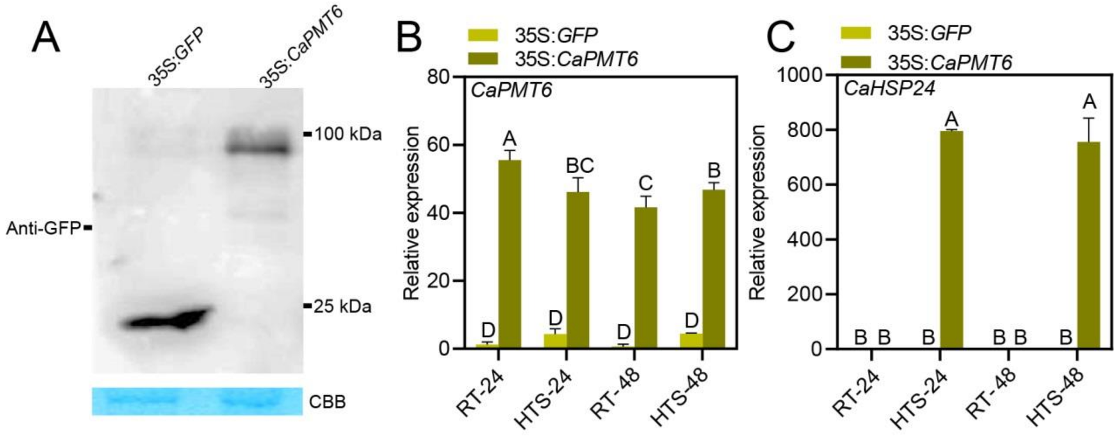

Figure S1. Transient overexpression of *CaPMT6* significantly upregulated *CaHSP24* in pepper plants. (**A**) The success of CaPMT6-GFP transient overexpression in pepper plants by western blotting using antibody of GFP, CBB = Coomassie brilliant blue. (**B**) The success of CaPMT6-GFP transient overexpression by RT-qPCR at 24 and 48 hpi. (**C**) *CaHSP24* was upregulated by transient overexpression of CaPMT6-GFP at 24 and 48 hpi in pepper leaves infiltrated with *A. tumefaciens* GV3101 cells harboring *35S:CaPMT6-GFP* or *35S:GFP*. In (**B** and **C**), *CaActin* was used as an internal control, the transcript levels of *35S:GFP* /RT-24 was set to 1, data are shown as means ± standard error of eight replicates, asterisks above the bars indicated significant differences among means ($P < 0.01$), as calculated with t-test.
